# Having a toilet is not enough: the limitations in fulfilling the human rights to water and sanitation in a municipal school in Bahia, Brazil

**DOI:** 10.1186/s12889-019-6469-y

**Published:** 2019-01-31

**Authors:** Édila Dalmaso Coswosk, Priscila Neves-Silva, Celina Maria Modena, Léo Heller

**Affiliations:** René Rachou Research Institute, FIOCRUZ-Minas, Av. Augusto de Lima 1715, Barro Preto, Belo Horizonte, MG 30190-002 Brazil

**Keywords:** Human rights, Water, Toilet, School, Hygiene

## Abstract

**Background:**

This article addresses the enjoyment of the human rights to water and sanitation (HRTWS), in particular access to toilets, in a public school in Bahia, Brazil.

**Methods:**

Participant observation of the school’s routine, focus groups with students in grades 8 and 9 of primary school (13 to 17 years old) and individual, semi-structured, interviews with members of school staff were applied, exploring access to water and sanitation by adolescent girls and boys.

**Results:**

Students and school staff reported that the amount of toilets was insufficient and that their conditions were often inadequate because they were plugged or dirty. The impact on girls is greater as toilets do not offer a clean and healthy environment for menstrual hygiene management. Several elements of the normative content of the HRTWS, especially accessibility, acceptability, quality, safety and dignity, were largely not fulfilled. The study identified that, to comply with the HRTWS, it is necessary to go beyond infrastructure, as the lack of maintenance; cultural elements and student participation hinder the usage of sanitary facilities. Since schools can be privileged spaces to train critical and reflective citizens and to foster autonomy and emancipation, education oriented by human rights and citizenship is an opportunity for a more equitable society. By increasing access to social, economic and cultural rights in all phases and aspects of life, including when children and adolescents are in a school environment, people are able to enjoy better living conditions and a higher standard of health.

**Conclusions:**

The study raised the importance of considering each community’s sociocultural aspects in analyzing access to sanitary facilities in schools, which are spaces where citizens’ rights should be exercised and fulfilled.

**Electronic supplementary material:**

The online version of this article (10.1186/s12889-019-6469-y) contains supplementary material, which is available to authorized users.

## Background

The recognition of the human rights to safe drinking water and sanitation (HRTWS) by the United Nations, in 2010 [1], impacts on the way that individuals and States deal with access to these services. Henceforth, access to water supply and sanitation services became a right that States must guarantee and cannot treat as charity or assistance.

The definition of the HRTWS comprises a series of normative content that must guide policies in the corresponding service sector: *availability*, sufficient quantities of water and toilets available during the day and night; *accessibility*, in a safe and physically accessible location; *affordability*, individuals must not devote a large portion of their family income to use these services to the point of impeding their access to other essential goods; *quality and safety*, with water of proper quality and hygienic toilets to avoid health risks. Toilets must be regularly cleaned and include handwashing facilities with soap; *acceptability*, toilets and water must respect local social and cultural characteristics; *dignity and privacy*, facilities must ensure privacy and dignity, allowing for menstrual hygiene management and disposal of those wastes [2, 3].

However, complying with the HRTWS is a complex process involving interests and, at times, contradictions on the part of both those responsible for fulfilling those rights (“duty bearers”) and those who possess rights (“rights holders”). In addition, sociocultural, economic and political aspects of particular locations interfere in efforts to fulfill rights and ensure access to water supply and sanitation services [4–6]. Therefore, local context must be considered if rights are to be ensured and made real.

Based on an empirical study in India that analyzed the relationship between local context and enjoyment of the HRTWS, Singh [4] concluded that works and services that aim to bolster the population’s HRTWS neglect many factors in their implementation and execution. In turn, time and the State’s resources are often wasted and access is not improved. The author highlights that aspects which reflect the sociocultural context of the communities receiving services are of extreme importance to realize the HRTWS.

A simplified understanding of the HRTWS would presume that these rights could be considered attained once a legal basis and infrastructure have been provided, regardless of the local context in the community where the implementation of these rights will, in fact, take place. Several authors remark that many infrastructure works aiming to improve access to water supply and sanitation are undertaken without incorporating the local cultures, knowledge and specific practices of each community. At the root of this is a techno-centric, imperative vision that deters intersectoral and interdisciplinary action, which can entail inadequate access [5–10]. In this sense, Singh [4] affirms that an understanding at the local level, where the HRTWS will be (or should be) fulfilled, is necessary to assess if the proposed measures will entail compliance or violation of these rights. For that author, there is an interface between duty bearers and rights holders, and understanding the gaps between those two contexts would help in realizing the HRTWS.

The relationship between adequate access to water supply and sanitation, hygiene practices and sickness, especially children with diarrhea, has been amply demonstrated [11–13]. Morgan et al. [14] highlight that schools are important places to reduce the burden of disease related to water supply and sanitation services as they are places where children, adolescents and adults spend a large portion of their day. Thus, availability of safe drinking water and sanitation services can determine the functioning of such institutions, since they are essential to maintain general cleanliness and for the concrete physiological needs of human beings.

Yet, despite ample knowledge of the importance of adequate sanitary services in schools for the health of children, adolescents and all individuals working in such environments, the last school census performed in Brazil, in 2017, shows that of all primary schools offering the primary education cycle, 6.1% do not possess a sanitation system, 52.3% possess a cesspit and only 41.6% are connected to a sewage network [15].

In addition to health problems, some studies demonstrate the importance of adequate sanitary facilities in schools for equity in access to education. Many girls do not attend school as they do not want to use mixed facilities or, in some cases, they avoid drinking water while they are attending school to deter the need to use the toilet [14, 16–19]. As per the findings of Sorenson et al. [20], reducing consumption of water can lead to dehydration and worse educational performance. In this regard, a study performed in Kenya by Freeman et al. [18] highlighted that improving water supply and sanitation services reduced the probability of absenteeism by girls by 58%.

The relevance of access to these services is recognized in goal 6 of the Sustainable Development Goals, particularly in target 6.2, which addresses access to sanitary facilities, paying special attention to women and girls. Without adequate access in schools, this target will not be achieved. In addition, goal 4 (to provide quality education) can also be mentioned. Especially target 4.1, which aims for equitable access for boys and girls; and target 4.a, aiming to improve education facilities that are disability and gender sensitive to provide safe, non-violent, inclusive and effective learning environments for all. These targets will also only be attained when schools offer universal access to sanitary facilities with respect for local cultures [21].

Despite the important relationship between health, education and sanitation, this subject is still neglected and studies aiming to assess access to this service are few. Understanding how the HRTWS are implemented and, consequently, conditions of access to water supply and sanitation services in schools can help to apprehend how local context influences implementation. It can also assist in identifying measures to make the HRTWS effective in this space. Against this backdrop, the present article assesses access to sanitation with a basis in the HRTWS in a public school in the State of Bahia, Brazil.

## Methods

Qualitative methods were adopted to perform the present research as they were considered the most appropriate tools for the study in question. Indeed, this approach allows one to understand or interpret phenomena through the meanings that people give to them [22]. Field work was carried out using the following methods: participant observation [23]; focus groups [24] with students using an interview script developed for this research (script in Additional file 1).; and individual semi-structured interviews [25] with the school’s principal and vice-principal using an interview script developed for this research (script in Additional file 2).

The research was carried out in a city in the extreme southern region of Bahia. The selected school is located in a neighborhood on the outskirts of the city that is known for its socioeconomic vulnerability. The school is the largest in the municipal region and its students originate from that neighborhood and others close by. According to data provided by the Municipal Secretariat of Education, in 2016, the school possessed 1.124 students and offered the entire curricula for primary education, from literacy training to youth and adult education (*Educação de Jovens e Adultos*), in all three trimesters. Moreover, the school was staffed with 59 teachers and 30 members of technical or general staff. The total number of students in the “primary education II” program (middle school) was 396, of which 81 students were in grade 8 and 53 in grade 9.

Participant observation was used to verify routines within the school and its structure in terms of water use and the HRTWS. This was carried out between October and December 2016 in the afternoon cycle. The data obtained contributed to creating the semi-structured schedule that was used to carry out focus groups with students and other individual interviews.

Students in grades 8 and 9 were selected to participate in this study as they were considered to have a more appropriate average age (13 to 17 years old) and level of maturity for the research in comparison to children in earlier grades. It was considered that most of the female students would have started menstruating, which would allow questions regarding female needs to be discussed. The groups were comprised of students who accepted to participate in the study after the project’s design was presented in their classroom. The focus groups were carried out in the school, were recorded with an audio recording device and were subsequently transcribed to a digital platform. The design of the focus groups was based on gender and age criteria, considering that the realization of HRTWS for adolescents is highly dependent on gender. Thus, homogeneous and heterogeneous groups, in terms of sex and school grades, were organized to obtain views from groups of only girls, only boys and both sexes together. Furthermore, some groups comprised peers from the same grade while others mixed students from different grades. Therefore, the four FG carried out were: FG01 (10 female participants from 9th grade classes); FG02 (10 female participants from 8th grade classes); FG03 (7 male participants from 8th and 9th grade classes); FG04 (9 female and 3 male participants from both grades).

The schedule used for the focus group with students sought to collect knowledge and opinions on: toilets and the normative content of the HRTWS; gender-related questions associated with toilets; who holds responsibility regarding the quality of access to those facilities; and the relationship between access to those facilities and economic, social and political matters in the community.

Semi-structured interviews were held with the principal and vice principal of the selected school, followed by the focus groups, in 2016. The interview with the school’s direction addressed: problems mentioned by the students; the opinions of students, teachers and other staff members with respect to the toilets; the normative content of the HRTWS; educational efforts to address appropriate hygiene, the use of toilets, and matters of personal and menstrual hygiene; human rights and particularly the HRTWS, and; training to address these subjects as a part of their professional activity. All interviews were recorded with an audio device and transcribed to a digital platform for analysis. After transcription, the collected data was analyzed using the technique of content analysis [26].

The normative content of the HRTWS [1] was taken into consideration to systematize participants’ comments: availability, accessibility, quality and security, acceptability, privacy and dignity. The normative content of affordability was not considered for the purposes of this research.

The research was approved by the Research Ethics Committee at René Rachou Research Centre under protocol CAAE 57950816.0.0000.5091 and by the Municipal Secretariat of Education. Data collection initiated after a presentation of the research to the school community. All participants signed an informed consent form and were informed that their participation was provided on a voluntary basis and their anonymity would be guaranteed.

## Results

The analysis was based on the data collected from the four focus groups performed with 39 adolescents (comprising 29 girls (14 from 8th grade and 15 from 9th grade) and 10 boys (6 from 8th grade and 4 from 9th grade)), the individual interviews with the school’s principal and vice-principal, and the participant observation of school routines. Those data were systematized using the normative content and the dimensions of the HRTWS and two analytical categories were established: “School Infrastructure and HRTWS” and “Beyond Infrastructure”.

### School infrastructure and the HRTWS

Within the examined environment, there are toilets and water available for cleaning and drinking. However, according to students’ reports, there are not enough toilets for all the students.

FG1: for the number of students that there are, I think that there should be more [toilets]. There should be more…sometimes when it gets really full, people have to wait…you know… “Let’s go, I need to go” [*laughter*].

In addition to toilets being insufficient in quantity, access to these services, mainly for students using wheelchairs is not adequate.

FG2: And it’s the cleanest. And students in wheelchairs don’t use this toilet. They use the one in the little building… They use the one in the little building because the little building has that thing for them to hold on to, and here it’s broken…

Toilet maintenance was also insufficient and they were, usually, broken and dirty. The lack of toilet paper leads to embarrassing situations and to dirtier toilets, as per the report of the focus group. Toilet paper was removed as it had led to the toilets becoming plugged, according to the staff and students. Moreover, there were no waste bins at the end of the year. In turn, this led to a vicious cycle of filth and failing hygiene, which created indecent conditions for the students and the cleaning staff. Also, as some doors can’t be closed, female students feel unsafe when using the toilet.

FG2: The door is broken too… Half of the doors are all broken… There’s no way of closing them… A friend holds the door for me…

FG3: Because there’s no door… The doors don’t have locks… Also… And the toilet is almost in front of the door …If you walk past it you can see inside … if you close it, the guys will lock you in…

Staff members assert that they are restricted by a lack of available resources from buying materials of better quality, such as waste bins, that are stronger and more suitable for long-term institutions hosting large numbers of people. Resources are also insufficient to repair those that are worn down from use, owing to play-fighting or occasional acts of vandalism. The toilets also did not provide adequate conditions for proper menstrual hygiene management.

FG1: Can I speak? Because sometimes the cleaning lady, at times, is disgusted at the thought of cleaning that toilet. All the blood there is, menstruation absorbent paper [sic] on the floor… And when you menstruate, you need to have toilet paper in the stall, right? Some girls are even too ashamed to take out the thing and they leave it there in the open, wrapped up in toilet paper and there it sits, in a, there’s not even a waste bin, so you have to put it somewhere. Do you leave it in the toilet? Some [girls] throw it in the toilet… or even on the floor.

In addition, handwashing stations with soap were not adequate, further hindering cleanliness and creating situations of risk for students’ health.

FG2: THERE HAS NEVER BEEN [*in unison*]. I think that since they constructed the bathroom they have never put soap there… How many years have I been studying here? Let me see, I think more than 8 years. I bring soap and toilet paper from home… Since my first day until today, I’ve never seen a bar of soap in all of primary school.

FG3: You end up coming out with your hands dirty and when you go to drink water, which you drink with your hand, or you have a snack… you just rinse your hand with water.

The number of sinks, according to the students, is largely insufficient for most male adolescent students. In the school’s bathrooms, there are three toilets and one handwashing station.

FG3: If 3 toilets are in use, you have to wait and the sinks down low… The sink at this height… and little faucets… They should be the same as the sinks in the shopping center…

The above commentary refers to the sink that, according to the student, is very low for most male adolescent students. In the school there is also one toilet and handwashing station adapted for wheelchair users.

FG3: Yes, but a wheelchair user can’t use it.

### Beyond infrastructure

It is important to highlight that the students did not like to use the school’s sanitary facilities and avoided using them at times.

FG1: I…I…I’ve not…I’ve not [gone] many times… And depending on the time, like, if it is almost time to leave, or if there are only two classes left before I can leave, I won’t go to the bathroom depending on what state it’s in… Also… I’ve already become used to not going through all of my classes. I go at home… Me, sometimes I’ve not gone through all of my classes… I’m used to not going to the bathroom.

This response was repeated in the three other focus groups. Participants commented that they only used the toilet if there is no other choice. Thus, untidiness and lacking hygiene and privacy can lead some students to use the toilet only when absolutely necessary.

FG1: I already have a problem. I have a loose bladder…Me too… a urinary infection.

Students also referred to problems related to the frequency with which the toilets were cleaned.

FG1: Well… there is a toilet. Clean though... The cleaners clean… They clean, they clean…They could clean more often… You know why? Because there are girls that go pee in the toilet and that go on the ground. They sit on the floor and go pee… They clean up real good, but then they go and … The flush is broken, there’s not even a seat cover to sit down on and pee.

During participant observation in the school, the daily routine was followed and it was possible to speak with students. On cultural and social matters that influence access to water and sanitation in the school, the vice principal was a key resource:

FG2: There are times when you go there and the bathroom is completely full, the bathroom at the end is locked sometimes. There are times that that they lock the bathroom. Because after 3:00 pm, the bathroom was clean, it was. Then they only clean at 5:00 pm or 5:20 pm for those [that come] at night… And at 5:00 pm too, that bathroom is already locked… It’s locked right up so that it’s clean for those [that come] at night… [*laughter*].

In addition, according to students’ reports, the period during which the bathroom is used is limited to the second class of the day until recess. Apparently, students are not allowed to leave the classroom during their first class of the day, and during the last class of the day the bathroom is cleaned and locked for students that will attend night classes. The common understanding is that during the first class of the day, students will have just arrived from home and will not need to use the bathroom. However, many students live at a considerable distance from the school. Additionally, during recess, the bathroom is very congested since the majority of students wait to use the toilet at the end of recess.

FG3: Something else… some people live far away… They only let us leave the class during the second class of the day. Students who live far away are going to get out… They can’t go, they have to go to class, sit in their seat… When they try to leave the teacher won’t let you… You wait the entire first class… During the fourth class after recess you can’t, then during the fifth class, in general, they are cleaning it so you can’t use it… You can only go during the second and third class, once you’ve started…

Owing to local cultural aspects and insecurity related to hygiene in bathrooms, girls and women normally do not sit down on the toilet and end up retaining their physiological needs for fear of contamination and diseases.

FG2: Because they don’t clean up… They find it disgusting, but there’s nothing disgusting, the women clean up everything properly… Really… Because of the state of the toilet, that there’s more filth in the toilet than on the floor… But some girls are filthy, aren’t they... Because after 3:00 pm, the bathroom has already been cleaned…

FG4: Ah, because some [girls] find it disgusting… What I don’t know, since they don’t touch the toilet… Right? [*girls laughing*] I don’t understand [*boy*]… Seriously [*girl*]… Because of pee… So that they don’t get any diseases… I think it’s that [they find it] disgusting too… The filth, who could manage to sit down?

The female students also reported that they spend the entire afternoon with the same absorbent pad, even though they believe it to be unhygienic to spend so many hours without changing it. They believe that it is riskier to change the absorbent pad in a dirty bathroom.

## Discussion

In the past few years, a considerable increase in the number of students attending the school assessed was observed due to the emergence of several residential buildings in neighboring areas. This is explained by the federal government housing program My House, My Life (*Minha Casa, Minha Vida*), which facilitates access to housing for families from lower classes. Nonetheless, the school did not undertake any renovations or further works, thus, maintaining the same number of toilets.

According to the Manual for School Building Performance [27], each school should have 1 toilet and 1 waste container for every 40 students. The number of female students in the school is greater than 40, yet despite them being the majority sex, female students possess the same number of toilets as their male counterparts. Consequently, there is unbalance with respect to the number of required services, which can be seen as noncompliance with the normative content of availability regarding access to sanitation services.

For each sex in the second cycle of primary education (middle school grades), there is one bathroom with four toilet stalls, one of which is adapted for wheelchair users. The national guidelines specify that 5 % of a school’s toilets must be suitable for persons with physical disabilities, that such toilets must be physically accessible, unobstructed and properly signed, and that a support bar must also be installed to allow the student to use the toilet independently [27]. Full conformity with these conditions was not observed in the school owing to the improper location of such toilets and the lack of signage for persons with visual disabilities. This constitutes further noncompliance with the normative content of accessibility.

Moreover, as the toilets’ physical conditions deteriorate throughout the year and maintenance is insufficient to keep all functioning correctly, the total number of available and operational toilets gradually decreases. More often, some are not kept in proper conditions (toilet plugged, stall door missing, toilet will not flush, among other issues). Privacy is not ensured owing to the lack of maintenance and timely repair of components that break throughout the year, such as doors and locks. Even if every toilet had a door of sufficient height, the position of the main door greatly exposes those that are inside the stall. This finding is similar to that of other studies, which indicate that students feel exposed in school bathrooms in Sweden [28]. In another study in France, 62% of students reported that they did not feel safe and 54% reported a lack of privacy [29]. This lack of privacy affects girls more, which can entail reduced participation in school or even reduced consumption of water whilst girls are in school to avoid using the toilet, as several studies have highlighted [14, 16–19].

In addition to problems regarding availability, accessibility and privacy, students reported that the toilets become very dirty. This affects other dimensions of the normative content of the HRTWS: acceptability, dignity, and quality/safety. These results coincide with other studies, including from developed countries. For example, in a public school hosting students from low-income groups in Melbourne, Australia, 73% of students stated that the toilets were not proper, 54% said there was not enough toilet paper and 42% said that they were not regularly cleaned [30]. Norling et al. [28], in England, and Hoarau et al. [29], in France, also obtained results indicating that the toilets were considered dirty or disagreeable.

This situation implies inadequate conditions for menstrual hygiene management, which directly affects girls’ dignity and health. According to other studies, a lack of proper guidance on the management of menstrual hygiene, water, sanitary facilities and hygiene-related materials, in low- and medium-income countries, creates problems for girls and limits their options in maintaining healthy personal hygiene during their menstruation [31, 32]. Consequently, barriers to menstrual hygiene in school can affect girls’ sense of dignity, well-being and involvement in school activities [31]. Pickering and Davis [33] highlight that a decrease in hygiene during menstruation can lead to infection of the reproductive system, pelvic inflammation and infertility. Therefore, inadequate conditions for menstrual hygiene management in bathrooms can turn such spaces into at-risk areas for female students’ health.

Another finding from the study showed that students face difficulties in adequately washing their hands. In accordance with the Manual for School Building Performance [27], there must be one handwashing station with a faucet for every 30 students and one soap distributor for every two handwashing stations, 5% of which should be suitable for wheelchair users. Considering the approximately 330 students in the school’s afternoon session, the bathrooms for each sex should have been equipped with at least six handwashing stations including their respective faucets [34]. Moreover, the handwashing station for wheelchair users was not signaled as such, and the faucet was not easy to use.

Many studies have identified that improving handwashing hygiene has the potential of reducing morbidity and mortality from infections transmitted by fecal-oral route or via direct contact, including gastrointestinal diseases and respiratory infections. It also reduces school absenteeism caused by related illnesses [35–39]. Furthermore, a WWAP report [36] reveals that four out of five people do not wash their hands after contact with urine and/or feces, which can lead to several diseases. For WHO [37], the simple act of washing one’s hands with water can substantially decrease the prevalence of diseases such as diarrhea, which is responsible for the death of 760.000 children under five years old every year worldwide.

Besides that, in a study performed by Bain et al. [38], it is estimated that 1.8 billion people in the world drink water contaminated with *Escherichia coli*, an indicator of fecal contamination Thus, improper water supply, sanitation services and hygiene are associated with considerable risks of diarrheal diseases [38]. The link between diarrhea and malnutrition, and the related effects of sanitation and hygiene, are still underestimated. Diarrhea is a significant cause of infant mortality that entails cognitive and economic effects associated with child malnutrition [40]. Thus, the school environment, where children spend most of their days, must have proper sanitary facilities to protect their health.

The problems revealed in this study are similar to other cases in which students have reported that they do not like to use their school’s toilet owing to problems such as disagreeable odors, bullying and insufficient facilities [28, 29]. Consequently, they avoid using toilets, which can create risks of developing diseases in the female genitourinary and intestinal tracts [29].

A systematic review of studies assessing the effect of water and sanitation in schools indicated that, in institutions where access to these services is precarious, a high prevalence of infectious, gastro-intestinal, neuro-cognitive and psychological diseases was observed [41]. Another study also pointed out the relationship between lacking access and psychosocial aspects, especially among women [42]. Thus, there is a verified need for adequate facilities not only to avoid infectious diseases, but also to reduce psychological problems that can befall girls, disrupting their participation in school and the mission to attain equity in schools.

Also, students experiencing problems with continence can suffer negative effects in their academic performance, as the majority of such students do not share their problems with their colleagues and teachers due to stigma and fear of bullying and social alienation. This creates challenges regarding the best way to support such youth, since they require unrestricted access to private and proper sanitary facilities throughout the school day [43].

Hence the significant impact of lacking policies on access to inclusive sanitary facilities and improved standards for toilets in schools. Addressing the challenges faced by youth with continence problems in schools could help to eliminate the barriers to successfully self-managing their symptoms. Self-management of continence problems requires a structured program of ingesting liquids and emptying the bladder. Inadequate sanitary facilities and restricted access make it difficult for youth to manage their incontinence [43]. Other health problems, such as intestinal issues that entail a heightened need to use the toilet, can cause the same effects.

Therefore, the bathroom appears to be invisible throughout the school’s day-to-day existence. This could perhaps owe to the fact that academic activities are given foremost attention and matters such as the physiological needs and human rights of those that have a right to education are ignored. For McIsaac et al. [44], the growing demands of the educational system on teachers and school direction staff limit the general support that a school could provide to health promotion activities.

In terms of the school’s routine organization, potential violations of the HRTWS were observed. Bathrooms are only cleaned before the beginning of the school day and before the end of the school day, for students attending night classes, and students only use the bathroom during recess. During classes, permission to use such facilities is required. This practice is common to many schools, as per indicated by other research sources [28–30]. This internal organization is established by the employees responsible for managing the establishment.

Therefore, this study, in addition to others, evidences the importance of infrastructure, but also of local context [4, 44, 45]. People have different ways of living and contexts within which knowledge is held. This leads to different interpretations of the same situations and events, which in turn produces different responses. This interferes in how situations develop in unpredictable and diverse manners [45]. For Long [45], “social interface situations are complex and multiple in nature, containing within them many different interests, relationships and modes of rationality and power”. The question of toilets in schools is a complex problem that requires multiple stakeholders and simultaneous interventions in order to attain a solution. McIsaac et al. [44] base themselves on an “understanding of the significance of deeply rooted traditions that cultivate unhealthy cultural norms and influence school communities”. Those authors highlight the contribution of community and organizational culture in supporting or obstructing health promotion in schools. Overcoming such challenges would come through implementing health promotion within schools and training people through community empowerment.

McIsaac et al. [44] emphasize the importance of leaders in schools, especially staff and their vital support with sufficient time and resources to prevail over potential indifference or resistance. Several studies [46–48] indicate that overcoming political and cultural barriers to health promotion would require schools to reflect on several factors: how to boost their organization capacity through partnerships; engaging the school community in decision making; establishing norms in the school; and “transform(ing) the culture of school so that health is embedded as the ‘way of life’ of school” [44]. Considering that human rights are essential to ensure proper health [49], it can generally be said that the culture of the school must be transformed so that human rights are embedded as the way of life of the school. Quality health and education could, thus, be attained.

According to the Law on the Rules and Guidelines of Brazilian Education [50] it is hoped that schools will prepare individuals to exercise citizenship. The exercise of citizenship is essential to upholding the democratic State and for enjoyment of civil, political, social, economic and cultural rights. Demanding the fulfilment of one’s rights and monitoring that process of realization is a part of exercising citizenship [51]. In this way, education is a right and, at the same time, an opportunity for other rights to be demanded and fulfilled.

Thus, the school can be a privileged space to train critical and reflective citizens, creating autonomy and emancipation [52–54]. But in order to do so, there must be clarity regarding the role of this space. Also, qualified professionals must be trained to work in human rights, using participative methodologies that lead to attitudes of active citizenship. In this way, individuals would demand enjoyment of their rights and monitoring of such enjoyment, but would also be conscious of their duties. An opportunity should exist to critically analyze contradictions in the school space and the living conditions, inequalities and injustices that students experience. Hence the importance of the school in preparing individuals to exercise citizenship; there is no social institution with comparable access to children and adolescents for such a long period of time that can fulfil this role.

Thus, education oriented by human rights and citizenship is an opportunity for a more equitable society. By increasing access to social, economic and cultural rights in all phases and aspects of life including when children and adolescents are in a school environment, people are able to enjoy better living conditions and a higher standard of health.

## Conclusion

International treaties create obligations for States to guarantee access to water supply and sanitation services based on the normative content of the HRTWS. However, this study indicates that the premises of the HRTWS are not being fulfilled in a school in the state of Bahia, Brazil.

Evidently, questions concerning context within the community receiving these services are of vital importance in realizing the HRTWS. Thus, consideration for local cultural factors can contribute to filling gaps and promoting effective access to these rights. In addition, it can help in critically analyzing themes that are invisible in everyday life, such as the use of toilets in schools and associated dynamics. This specific case can allow the school in question to reflect on its social functions and its role as a space for citizen education.

Ensuring access to water supply and sanitation in schools can promote health in schools. It contributes to creating a healthy environment and preventing diseases, mainly for girls, who are most affected by substandard services. Critically analyzing conditions of access to these services in the school environment, together with students, can contribute to their education as citizens who are conscious of their rights, related policies, structural dynamics and power relations. Above all, it would enlighten them on educational questions and the role of the school in preparing emancipated individuals that can contribute to making the social changes required for a fairer society with respect for human rights, health and quality education.

## Additional files

Additional file 1: Interview script for the focus group with students. (DOCX 15 kb)

Additional file 2: Individual interview script with the school’s principal and vice-principal. (DOCX 14 kb)

## Abbreviations

HRTWS: Human Rights to Water and Sanitation
