# Supplementary material for: Having a toilet is not enough: the limitations in fulfilling the human rights to water and sanitation in a municipal school in Bahia, Brazil
Source: BMC Public Health. 2019 Jan 31;19:137. doi: 10.1186/s12889-019-6469-y (PMC6357509; doi:10.1186/s12889-019-6469-y)
Supplement: Supplementary file 1 — Interview script for the focus group with students. (DOCX 15 kb) [file 12889_2019_6469_MOESM1_ESM.docx]

**ADDITIONAL FILE 1 - INTERVIEW SCRIPT FOR THE FOCUS GROUP WITH STUDENTS**

Interview script – 8^th^ and 9^th^ students – 4 focus groups.

1. Do you consider the availability of drinking water and for proper hygiene and cleaning purposes in your home? What are the reasons for this opinion?
2. Do you consider the quantity, privacy, and dignity of the bathrooms adequate? What reasons justify this opinion?
3. Is there soap and water available in the student's bathroom every day? What influence does it have?
4. Do you consider drinking water and sanitation ideal in school? What reasons justify this opinion?
5. Have you had the opportunity to think about and discuss HRTWS in school? In what situation?
6. Do you think there is a difference in the needs of boys and girls in relation to the use of the bathroom in the school? Why?
7. Is there any situation where the lack of water in school makes you missing classes?
