# Supplementary material for: Having a toilet is not enough: the limitations in fulfilling the human rights to water and sanitation in a municipal school in Bahia, Brazil
Source: BMC Public Health. 2019 Jan 31;19:137. doi: 10.1186/s12889-019-6469-y (PMC6357509; doi:10.1186/s12889-019-6469-y)
Supplement: Supplementary file 2 — Individual interview script with the school’s principal and vice-principal. (DOCX 14 kb) [file 12889_2019_6469_MOESM2_ESM.docx]

**ADDITIONAL FILE 2 - INDIVIDUAL INTERVIEW SCRIPT WITH THE SCHOOL’S PRINCIPAL AND VICE-PRINCIPAL**

Interview script – School’s principal and vice-principal.

1. Is the water supply continuous? It is sufficient for the demands of school?
2. Does the school's Political Educational Project (PEP) foresee the direct or indirect discussion of human rights? What about HRTWS? Are there other documents that directly or indirectly refer to the right to water?
3. Are the amount, privacy, dignity and accessibility of restrooms in the school suitable for students, teachers and other staff? What reasons justify this opinion?
4. Is there any form of instruction for proper hygiene and toilet use? Who is responsible for this activity? And the issues of intimate and menstrual hygiene?
5. Have you had any specific training that facilitates handling these issues in your professional activity?
